# Preoperative walking recommendation for non-cardiac surgery patients to reduce the length of hospital stay: a randomized control trial

**DOI:** 10.1186/s13102-021-00317-w

**Published:** 2021-07-28

**Authors:** Olga L. Cortés, Mauricio Herrera-Galindo, Claudia Becerra, Mónica Rincón-Roncancio, Camilo Povea-Combariza, Maribel Esparza-Bohorquez

**Affiliations:** 1 Research Unit and Nursing Department, Fundación Cardioinfantil-Instituto de Cardiología, Cl. 163a #13B-60, Bogotá D.C, Colombia; 2 Faculty of Health Sciences, Universidad Autónoma de Bucaramanga, Avenida 42 No 48-11PBX, Bucaramanga, Colombia; 3 Nursing Department, Fundación Cardioinfantil-Instituto de Cardiología, Cl. 163a #13B-60, Bogotá D.C, Colombia; 4 Cardiovascular Rehabilitation Department, Fundación Cardioinfantil-Instituto de Cardiología, Cl. 163a #13B-60, Bogotá D.C, Colombia; 5 Faculty of Medicine, Universidad Nacional de Colombia, Cra 45, Bogotá D.C, Colombia; 6 Nursing Department, Fundación Oftalmológica de Santander-Clínica Carlos Ardila Lulle, FOSCAL, Calle 155A No23-60, Floridablanca, Colombia

**Keywords:** Non-cardiac surgery, Walking, Preoperative time, Length of hospital stay, Clinical trial

## Abstract

**Background:**

Even though the importance of preparing patients for a surgical event is recognized, there are still gaps about the benefit of improving functional capacity by walking during the waiting time among patients scheduled for non-cardiac surgery. The aim of this study was to evaluate the impact of pre-surgical walking in-hospital length of stay, early ambulation, and the appearance of complications after surgery among patients scheduled for non-cardiac surgery.

**Methods:**

A two-arm, single- blinded randomized controlled trial was developed from May 2016 to August 2017. Eligible outpatients scheduled for non-cardiac surgery, capable of walking, were randomized (2:1 ratio) to receive a prescription of walking 150 min/week during the whole pre-surgical waiting time (n = 249) or conventional care (n = 119). The primary outcome was the difference in hospital length of stay, and secondary results were time to first ambulation during hospitalization, description of ischemic events during hospitalization and after six months of hospital discharge, and the walking continuation. We performed an intention to treat analysis and compared length of stay between both groups by Kaplan–Meier estimator (log-rank test).

**Results:**

There were no significant differences in the length of hospital stay between both groups (log-rank test *p* = 0.367) and no differences in the first ambulation time during hospitalization (log-rank test *p* = 0.299). Similar rates of postoperative complications were observed in both groups, but patients in the intervention group continued to practice walking six months after discharge (*p* < 0.001).

**Conclusion:**

Our study is the first clinical trial evaluating the impact of walking before non-cardiac surgery in the length of stay, early ambulation, and complications after surgery. Prescription of walking for patients before non-cardiac surgery had no significant effect in reducing the length of stay, and early ambulation. The results become a crucial element for further investigation.

*Trial registration*: PAMP-Phase2 was registered in ClinicalTrials.gov NCT03213496 on July 11, 2017.

## Background

Globally, every year 200-millions of major non-cardiac surgeries are performed, mostly in adult populations [1, 2]. Between 2004 and 2012, there was a 38.2% increase in these surgeries (312 million/year), especially in developing countries [2]. In 2012, the global rate of general surgery was 4,469 operations per 100,000 people, which increased the total health expenditure by USD 400 USD per capita [1]. In the United States, in 2010, ten million non-cardiac surgeries were performed because of musculoskeletal disorders (84%), neoplasia (61.4%), lesions due to accidents (43.2%), and digestive diseases (36.2%), being the most prevalent problems [3]. Notably, cardiovascular complications after non-cardiac surgery happen within the first 30 days. These complications have affected more than 10 million people worldwide, with a mortality rate of around 1.5% [1].

One factor related to complications after these surgeries has been a sedentary lifestyle [3]. The prevalence of physical inactivity of adults scheduled for non-cardiac surgery was about 59% in the Promoting ambulation project (PAMP) study phase I [4]. Inactivity may be a secondary consequence of the disease that has triggered the need for the surgery, or it may be related to other factors such as age and comorbidities that may determine the inability to perform daily living activities [5]. Preoperative variables such as low functional status present in sedentary patients have been associated with prolonged length of stay, and patients who stay longer in the hospital, have the worse clinical outcomes [8]. In this way, identifying that low functional status is a factor related to prolonged LOS in patients of scheduled non-cardiac surgery, promoting interventions such as walking, have the potential to reduce LOS also impacting hospital outcomes like costs, quality access, efficiency, and equity in hospital care [8].

However, despite knowing about the impact of exercise and physical activity on health outcomes [6, 7], there is a lack of information about the efficacy of a preoperative walking recommendation for non-cardiac surgery to reduce the length of stay (LOS) [6, 7]. The results about the impact of aerobic exercises, such as walking, are discordant concerning their impact and length of hospital stay when applied to patients before non-cardiac surgery. In the meta-analysis developed by Hughes (2019), in which the impact of aerobic exercise was evaluated in clinical trials, the investigators showed a reduction in the result in global morbidity and lung morbidity. Nevertheless, it did not show differences in the length of hospital stay or the evaluation of the post-surgical walk evaluated through the Six-minute walk test. These results of investigators interpret these results as the need to continue exploring the relationship between exercise patients before surgery and LOS. [9].

Accordingly, this study sought to evaluate the impact of the recommendation for walking a minimum of 150 min/week during waiting time before surgery compared with a control group in the length of hospital stay and early ambulation among adults scheduled for non-cardiac surgery. Likewise, this study aimed to describe the clinical complications during hospitalization and 30 days after surgery. We also reported the frequency of continuation of the practice of walking at six months after surgery.

## Methods

This clinical trial study is reported according to CONSORT guidelines/methodology [9]. The study was registered retrospectively at Clinicaltrials.gov, NCT03213496 on July 11, 2017. We confirm that all methods were performed following the relevant guidelines and regulations regarding the protection and patient safety evaluated by the Ethics Committee in Research from FOSCAL clinic (Comité de Ética en Investigación CEI-FOSCAL) and the Ethics Committee in Clinical Research from Fundación Cardioinfantil Instituto de Cardiología (Comité de Ética en Investigación Clínica CEIC-IRB00007736). This study was considered of minimal risk by both Committees. All participants signed an informed consent form before the process of randomization.

### Study design

The “Promoting ambulation project (PAMP) Phase II was a parallel-group, open-label, randomized controlled clinical trial including patients aged ≥ 30 years, scheduled for non-cardiac surgery. Participants were assigned [ratio 2:1] to receive a structured prescription of 150 min weekly of walking before surgery along with all of their waiting time before surgery or usual care [Not receiving a recommendation of walking].

### Setting

We recruited patients from two fourth-level university hospitals from two cities of Colombia that performed the study between May 10, 2016, to August 31, 2017; and the study protocol had approval by the Ethics Committee from both hospitals (Fundación Cardioinfantil Instituto de Cardiología, from Bogotá, and Clínica FOSCAL, Bucaramanga).

### Inclusion and exclusion criteria

Patients were eligible if they were adults (≥ 30 years), were scheduled for non-cardiac surgery under general or regional anesthesia (spinal or epidural), were able to mobilize upon admission, had to remain in the hospital for a minimum of 24 h after surgery, and were able to sign a consent form. We excluded patients with motor disabilities, state-of-consciousness alterations, uncontrolled chronic pain, and whose pre-surgical waiting time was known to be less than one week.

### Sample size

For this study's primary purpose, we considered a minimally significant difference to be one day of hospital stay. Based on our previous research, we estimated the mean hospital stay after non-cardiac surgical procedures at 2.2 (SD = 1.1) and 3.3 (SD = 0.8) days for the categories of early and late discharge, respectively (Cortés OL., 2018). Thus to detect a mean difference of one day of stay, conservatively considering a standard deviation of 2–3 days, with 90% statistical power (type I error probability = 0.05), under a 2:1 randomization ration, the minimum sample size required was 368 patients (249 assigned to the intervention vs. 119 assigned to the control group).

### Randomization and blinding

**Fig. 1:**
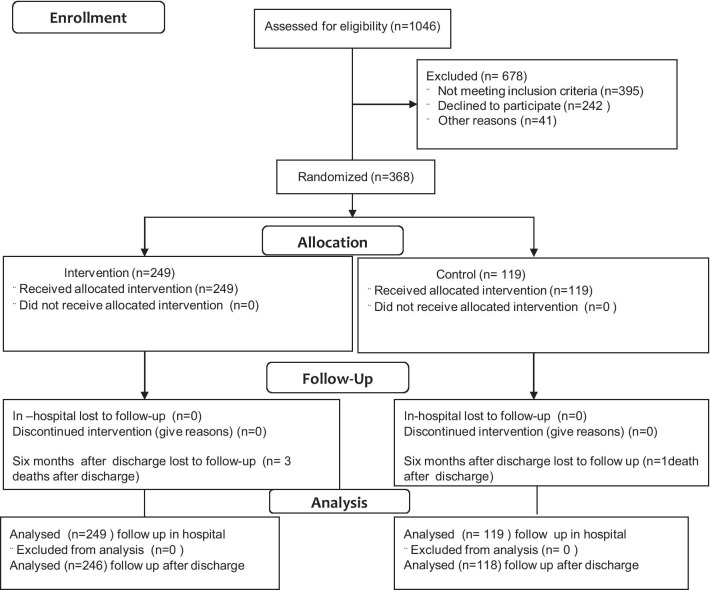
Consort flow diagram

Randomization was performed using a computerized random number generator centralized at Fundación Cardioinfantil. Patients were allocated to an intervention group (walking prescription/accelerometer) or a conventional care group (without walking recommendation/accelerometer) (Fig. 1). The patient allocation was kept by using sealed envelopes administered by a person not related to recruiting. Patients in the intervention group were assessed by one physiatrist doctor and also by a sports physician to prescribe the exercise and install the accelerometer. The surgeons or any physician provider of care of each patient, researchers, and analysts were blind regarding the assignation during pre, trans, and post-operatory time.

### Intervention and procedures

Patients were recruited from the outpatients' list scheduled daily for non-cardiac surgery from each study site by a nurse coordinator of the study at each hospital. Eligible patients were approached at the end of the appointment with the surgeon in each of the centers and invited to participate in the study and, upon knowing the aims of the study and acceptance to participate, underwent randomization after signing a consent form in an individual interview.

This randomization included also a randomly assignation of twenty patients by each group to carry an accelerometer out to evaluate METs-min/week [metabolic equivalents], during the waiting time, as a way to validate walking adherence and determine differences between both groups.

Once patients signed the informed consents were evaluated based upon their demographic and medical history and also about baseline physical activity by a physician. This evaluation was carried out by two different doctors in different offices in order to avoid the risk of contamination of the intervention. All patients had to completed the International Physical Activity Questionnaire (IPAQ-short) (Guidelines for the data processing and analysis of the “International Physical Activity Questionnaire, 2009) [10]. Using the IPAQ-short, they classified according to their physical level of activity as active (1,500–3,000 METs-min/week), with moderate exercise (600–1,499 METs-min/week), or sedentary (< 600 METs-min/week).

Each patient allocated to the intervention group received individual walking instructions, face to face both verbally and in writing with a prescribed form, by a physician and a nurse. The education included walking at least 150 min/week, divided into sessions of 50 min daily at least three days/week, after warming up and stretch, to be performed throughout the whole pre-operatory waiting period. These patients also received instruction to complete a walking week-diary at home. They also received weekly-reinforcement calls to promote activity adherence before surgery. Patients in the control group did not receive any walking instruction.

We obtained and completed information from the patients' clinical variables (demographic, health antecedents, indication, type of surgery, evolution, complications, time to the first ambulation during hospitalization, and length of stay) retrospectively and collected the information from patients' diary provided on the surgery date. We contacted patients by phone calls after six months (week 12) after discharge. Patients from both groups were interviewed about post-surgical complications. Furthermore, patients in the intervention group were interviewed if they continued performing the doctor's prescription, and patients in the control group were asked about themselves in the present time they were performing any exercise, including walking.

### The accelerometer

The equipment that was used to measure energy expenditure was the accelerometer actiheart® (CamNetech Ltd, UK). This device was implemented given its capacity to capture both the displacement in the three axes and simultaneously the heart frequency to obtain the energy expenditure (METs) accomplished with a minimum of 6 consecutive days of permanent use. For the analysis of the results, the accelerometer was recorded between days 1 to 6. The data were captured and processed by the analyst of the study, blinded to the assignment of the intervention. The information for each accelerometer was integrated into a program with tools for data analysis [StataCorp. 2011.Stata Statistical Software:Release 12.College Station, TX:StataCorp LP] This program allowed data analysis to be sent to the project data analyst and then be integrated into the rest of the analysis.

### Study outcomes

The primary outcome was the difference in length of hospital stay in days/hours. The secondary outcomes were time first to walk (ambulation) after surgery while in hospital and the frequency of ischemic events during hospitalization (acute myocardial infarction [AMI], cerebrovascular accident [CVA], deep venous thrombosis [DVT]), falls, and all causes of death. Events were assessed up to six months after discharge, including the continuity of walking for the intervened group and the initiative of walking for the conventional group.

### Statistical analysis

The data analysis was performed using StataCorp.2011. Stata Statistical Software: Release 12. College Station, TX: StataCorp LP. Continuous variables were described using means (standard deviation) or medians (interquartile range) when non-normally distributed. Alternatively, we performed a logarithmic transformation to reduce said asymmetry. Discrete variables were described as counts (percentages). The Student's t-test was used to evaluate differences in means of continuous variables between study groups or the non-parametric Kruskal–Wallis test to contrast variables whose distribution was not expected. We used the chi-squared test or the Fisher's exact test to assess differences in the distribution of discrete variables whenever the count expected in any cell of the contingency tables was < 5 observations. Finally, a time-to-event analysis was performed, employing the Kaplan–Meier estimator and the log-rank test to determine differences in the hospital stay or the first walk between the intervention and the conventional group [11].

## Results

### Participants

Of the total number of patients included (n = 368), all completed the study (Fig. 1). There were no group differences on demographics, medical history at other experimental characteristics and control groups, indicating baseline comparability. There were minor differences related to socioeconomic status (Tables 1, 2).

**Table 1:** Description of demographic characteristics of participants in the baseline

Characteristic	Intervention n = 249 (%)	Control n = 119 (%)	*p* value
Age in years, mean (SD)	59 (11.8)	59 (13.0)	0.970
Male	124 (49.8)	51 (42.9)	0.212
Level of schooling			
Elementary	49 (19.6)	30 (25.2)	0.349
High school	62 (24.9)	34 (28.6)	
Technology/university	138 (55.5)	55 (46.2)	
Occupation			
Employed	66 (26.5)	31 (26.0)	0.990
Independent worker	74 (29.7)	37 (31.1)	
Retired/unemployed	109 (43.8)	51 (42.9)	
Socioeconomic status			
1–2 (Low)	57 (22.9)	44 (37.0)	0.018
3–4 (Intermediate)	160 (64.3)	62 (52.1)	
5–6 (High)	32 (12.8)	13 (10.9)	
Marital status			
Married	155 (62.3)	63 (52.9)	0.445
Common-law	37 (14.8)	21 (17.7)	
Single, widowed, or divorced	57 (22.9)	35 (29.4)	
Origin			
Urban	236 (94.8)	110 (92.4)	0.375
Rural	13 (5.2)	9 (7.6)	

Values in each cell correspond to means (standard deviation), counts (proportions), or median [interquartile range].

*SOAT* Obligatory Traffic Accident Insurance

**Table 2:** Description of the health antecedents of participants in the baseline

Medical antecedents	Intervention (n = 249)	Control (n = 119)	*p* value
Family health history, n (%)			
Arterial hypertension	125 (50.2)	59 (49.6)	0.911
Diabetes	90 (36.1)	31 (26.1)	0.054
Dyslipidemia	67 (26.9)	27 (22.7)	0.385
Arrhythmia	20 (8.0)	5 (4.2)	0.172
Peripheral vascular disease	42 (16.9)	15 (12.6)	0.290
Transient ischemic attack	9 (3.6)	5 (4.2)	0.776
Cerebral vascular disease	29 (11.7)	9 (7.6)	0.229
Myocardial infarction	68 (27.3)	24 (20.2)	0.139
COPD	15 (6.0)	10 (8.4)	0.396
Active cancer	98 (39.4)	53 (44.5)	0.345
Cardiovascular risk, n (%)			
Current smoking	7 (2.8)	6 (5.0)	0.365
Arterial hypertension	93 (37.4)	44 (37.0)	0.945
Dyslipidemia	46 (18.5)	23 (19.3)	0.844
Diabetes mellitus	22 (8.8)	8 (6.7)	0.488
Cardiovascular disease, n (%)			
Arrhythmia	13 (5.2)	6 (5.0)	0.942
Peripheral vascular disease	29 (11.7)	12 (10.1)	0.656
Transient ischemic attack	4 (1.6)	4 (3.4)	0.279
Cerebral vascular disease	0 (0.0)	1 (0.8)	0.323
Myocardial infarction	9 (3.6)	3 (2.5)	0.758
Other comorbidities, n (%)			
COPD	4 (1.6)	2 (1.7)	1.000
Cancer	53 (21.3)	38 (31.9)	0.027
Total prior surgeries	2.0 [3.0]	2.0 [2.0]	0.066
Medications taken chronically before surgery, n (%)			
Antihypertensive	93 (37.4)	46 (38.7)	0.809
Beta-blocker	16 (6.4)	12 (10.1)	0.216
Statins	50 (20.1)	17 (14.3)	0.179
Diuretics	10 (4.0)	11 (9.2)	0.043
Gastric mucosal protectors	20 (8.0)	11 (9.2)	0.695
Prophylactic antithrombotic agent	27 (10.8)	17 (14.3)	0.341

Values in each cell correspond to means (standard deviation), counts (proportions), or median [interquartile range]

*COPD* Chronic obstructive pulmonary disease, *NSAI* non-steroidal anti-inflammatory

Related to physical activity assessment at baseline, the study population showed a Body Mass Index (BMI) over 26 (SD 3.4) and a higher prevalence of sedentary-moderate levels of physical activity (83.3–85.6%) in both groups (Table 3). Regarding the type of surgical intervention, patients in the intervention and the control group required hospitalization for more than one night (87.1% vs. 93.3%, *p* = 0.077), and a small proportion was discharged earlier than planned initially. The more prevalent length of stay was similar for both groups, and it was around two days for the intervention and the control group, respectively (63.0% vs. 71.4%) (Table 4).

**Table 3:** Findings of physical exam upon admission and level of physical activity in the baseline

Characteristic	Intervention (n = 249)	Control (n = 119)	*p* value
Assessment upon admission (SD)			
Heart rate (bpm)	74.2 (10.5)	73.1 (10.5)	0.200
Systolic blood pressure (mmHg)	120.6 (16.4)	118.4 (14.9)	0.246
Diastolic blood pressure (mmHg)	71.9 (9.2)	71.1 (9.7)	0.606
Breathing frequency (rpm)	17.4 (2.3)	17.2 (2.1)	0.250
Body mass index (kg/m^2^)	26.7 (4.0)	26.1 (3.4)	0.215
Waist-hip index	0.94 (0.10)	0.93 (0.13)	0.574
Physical activity-patient perception, n (%)			
Frequent	65 (26.1)	35 (29.4)	0.753
Occasional	105 (42.2)	46 (38.7)	
Sedentary	79 (31.7)	38 (31.9)	
METS on admission (IPAQ)	721.5 [858.5]	796.0 [834.0]	0.157
Level of physical activity (IPAQ), n (%)			
Sedentary	109 (43.8)	46 (38.7)	0.626
Moderate	104 (41.8)	53 (44.5)	
Active	36 (14.5)	20 (16.8)	

Values in each cell correspond to means (standard deviation), counts (proportions), or median [interquartile range].

IPAQ: International Physical Activity Questionnaire

**Table 4:** Description of characteristics of surgery and procedures performed during hospitalization

Characteristic	Intervention (n = 249)	Control (n = 119)	*p* value
Surgical waiting time (mean *days, min and max*)	15 [3–233]	17 [3–378]	0.668
Surgical time hour: min (RI)	1:42 (1:29)	1:54 (1:30)	0.209
Length of stay days, n (%)			
< 1	30 (12.1)	8 (6.7)	0.188
1–2	62 (24.9)	26 (21.9)	
> 2	157 (63.0)	85 (71.4)	
Type of surgery, n (%)			
Surgery modified to ambulatory	32 (12.9)	8 (6.7)	0.077
Required hospitalization for more than 24 h	217 (87.1)	111 (93.3)	
Scheduled surgery, n (%)			
General surgery	60 (24.1)	25 (21.0)	0.296
Urology	101 (40.6)	56 (47.1)	
Gynecology	41 (16.5)	22 (18.5)	
Orthopedic	30 (12.0)	6 (5.0)	
Other specialties	17 (6.4)	10 (8.4)	
Type of anesthesia, n (%)			0.603
General	195 (78.3)	87 (73.1)	
Regional	50 (20.1)	30 (25.2)	
Other	2 (1.7)	4 (1.6)	
Type of wound, n (%)			0.083
Clean	83 (33.3)	30 (25.2)	
Clean contaminated	166 (66.7)	88 (73.9)	
Contaminated	1 (0.8)	0 (0)	
Type of incision, n (%)			0.575
Open	152 (61.0)	69 (58.0)	
Laparoscopy	97 (39.0)	50 (42.0)	
Complications during hospitalization, n (%)			
Death	1 (0.4)	0	
DVT (Deep Venous Thrombosis)	1 (0.4)	0	
Fall	1 (0.4)	0	
Complications six months after the surgery, n (%)			
Death	3 (1.2)	1 (0.8)	0.749
DVT (Deep Venous Thrombosis)	2 (0.8)	0	0.328

Values in each cell correspond to means (standard deviation), counts (proportions), or median [interquartile range]

### Study outcomes

#### Description of events

There were few cardiovascular events during hospitalization and after discharge. There was one death during hospitalization, one DVT, one fall in the intervention group, and no control group events. Six months after hospital discharge, there were two DVT (0.8%) and three deaths in the intervention group (1.2%), and one death in the control group (0.8%) (Table 4).

#### Before surgery walk validation

**Fig. 2:**
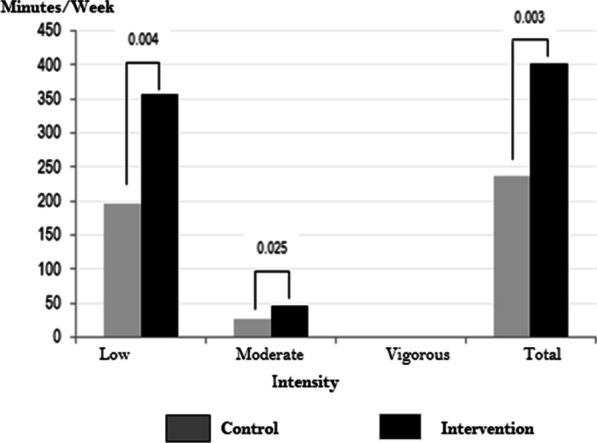
Time median (minutes/week) of physical activity measured using accelerometers, by intensity and group. Estimations are based on 20 patients for each group. Intensity: low (1.5—3.0 MET), moderate (3.1—6.0 MET), and vigorous (6.1—9.0 MET). *p* values estimated were obtained via the Kruskal–Wallis test

The median surgical waiting time was about 15 days (IQR 3–233) for the intervention group and 17 days (IQR 3–378) for the control group, with no significant differences (Table 4). The energy expenditure during physical activity (METs/hr.) median differences between both groups using accelerometers showed significant differences. Patients in the intervened group walked 403 [IQR 268; 630] min/week, whereas patients in the control group walked 237 min/week [IQR 200; 321] (*p* = 0.003). These differences remained for individuals with low and moderate walking intensities (1.5–3.0 MET, *p* = 0.004; 3.1–6.0 MET *p* = 0.025), respectively (Table 5, Fig. 2).

**Table 5:** Description of physical activity before surgery, during hospitalization, and after discharge

Characteristic	Intervention (n = 249)	Control (n = 119)	*p *value
Activity validation before surgery			
Accelerometer users	20 (8.4)	20 (16.8)	–
Physical activity (min/week)			
Low (1.5–3.0 MET)	358 [230–507]	196 [166–255]	0.004
Moderate (3.1–6.0 MET)	45 [38–86]	26 [4–47]	0.025
Vigorous (6.1–9.0 MET)	0 [0–1]	0 [0–1]	0.952
Total	403 [268, 630]	237 [200, 321]	0.003
Time to first walk after surgery in hospital (hours), n (%)			
Walk before to 24 h	153 (61.4)	68 (57.1)	0.710
Walk between 24 and 72 h	88 (35.3)	47 (39.5)	
Walk time over 72 h	8 (3.2)	4 (3.4)	
Place of first ambulation, n (%)			
Around the bed	13 (5.2)	7 (5.9)	0.218
From the bed to the bathroom	164 (65.9)	87 (73.1)	
From the bed to the hallway	35 (14.1)	9 (7.6)	
Through the hallway	7 (2.8)	6 (5.0)	
Other	30 (12.0)	10 (8.4)	
Following walking behavior after six months from the surgery			
Were you walking after surgery at least 150 min at the week? Yes	191 (76.7)	44 (36.9)	< 0.001

*METS* is defined as the amount of oxygen consumed while sitting at rest and is equal to 3.5 ml O_2_ per kg body weight × min

#### During hospitalization and after hospital discharge

**Fig. 3:**
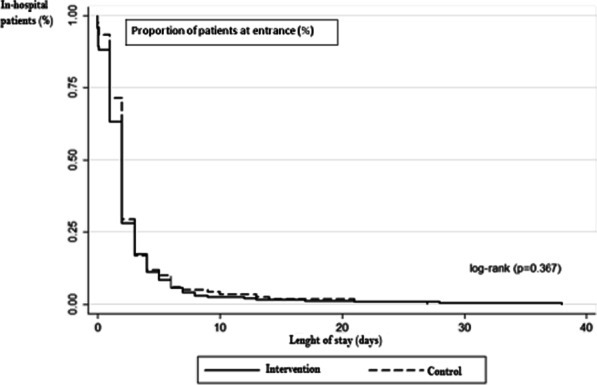
Time at discharge (of hospital stay) in the intervention and control group. No differences were noted in the time of stay between the groups. (log-rank test *p* = 0.367)

**Fig. 4:**
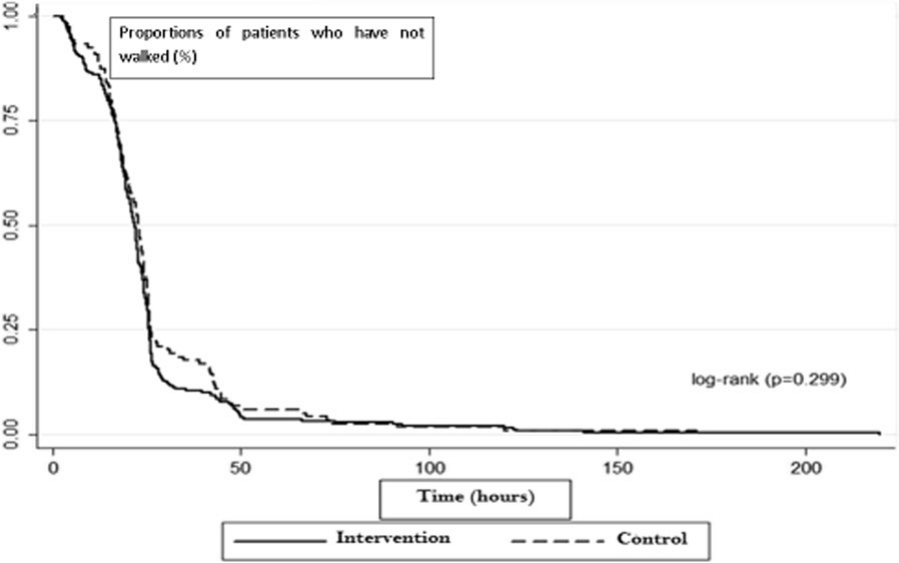
Time to the first ambulation during hospitalization of intervention and control group. No differences were noted in the first walk during the post-operatory between both groups (log-rank test *p* = 0.299)

Although we observed that a smaller fraction of patients in the intervened group remained hospitalized compared with the control group (63.1% vs. 71.4%, *p* = 0.188) (Table 4), there were no statistically significant differences in length of hospital stay (log-rank test *p* = 0.367). Likewise, there were no significant differences in the beginning ambulation during the post-operatory period between both groups (log-rank test *p* = 0.299) (Figs. 3, 4). After surgery, the first ambulation was more often observed between the bed and the bathroom in the in the intervention group (65.9%) than in the control group (73.1%).

After six months of hospital discharge we identified significant differences (Chi square) in walking behavior. Patients in the intervention self-reported they continue walking according to the doctor's prescription before surgery. They were more likely to continue walking [76.7%, n = 191 of 243, 95% CI 164 to 220.09] than patients in the conventional group [38.0%, n = 44 of 118, 95% CI 31.9 to 59.06)] (*p* < 0.001).

## Discussion

In this trial, the prescription of walking 150 min each week during the waiting time, compared with conventional care for adults going to non-cardiac surgery, did not reduce the length of stay or the time to the first ambulation of patients during hospitalization. Similar rates of postoperative complications were observed in both groups. However, significantly patients in the intervention group self-reported continue to practice walking six months after discharge.

A meta-analysis of data from trials involving 347 patients scheduled for cardiac surgery, aerobic exercise before surgery showed a significant reduction in length of stay of 3.2 days (mean − 3.2, 95% IC − 5.73, − 0.69] [6]. Being consistent with our results, two meta-analyses of aerobic exercise before general surgery have shown no impact on hospital stay length. Moran et al. [12] developed the systematic review, including 435 patients (from randomized controlled trials) undergoing intra-abdominal surgery, evaluated the effect of inspiratory muscles training, aerobic exercise, and resistance training before surgery in outcomes post-surgery [12]. It showed a reduction of complications (odds ratio 0.59, 95% CI 0.38, 0.91, *p* = 0.03), but it could not show differences in length of stay (mean − 1.62, 95% CI − 75, 4.3 days). A recent meta-analysis of data from trials involving 927 patients scheduled for gastrointestinal surgery related to cancer, the prehabilitation programs, including aerobic exercise, showed no differences between groups related to complications after surgery, mortality, and length stay [13].

In the non-surgical setting, aerobic exercise has proved to produce positive changes in cardiorespiratory function and physical performance in general populations and populations at risk, such as the elderly [14, 15]. However, in our study, walking before non-cardiac surgery did not show differences in length of stay, early ambulation post-surgery, or any impact associated with complications. We offer some potential explanations for these findings. First, walking is frequently prescribed as part of patients' physical preparation before general surgery, although there is a lack of evidence to demonstrate its aerobic benefits [7, 24]. Exercise modifies body composition, cardiovascular fitness, flexibility, muscular endurance, and strength and improves physical activity by 85%, as it has been demonstrated in solid research-based in healthy individuals and patients going to cardiovascular surgery. However, there is a low quality of evidence [7] about the impact that may provide unsupervised walking before surgery in patients´ outcomes during hospitalization and after discharge, mainly when this evidence is based on indirectness [respiratory exercises, cycling, but no unsupervised walking] and inconsistency of the results of the studies [24].

Second, walking intervention, which can be performed by patients at home or in an unsupervised way, represents a low- moderate-intensity aerobic exercise that may not improve its components within a variable short time as it is the surgical waiting time (median of 15 days in our study). Third, given that walking provides low-moderate intensity as an aerobic exercise, in some other studies, this intervention was being provided to the participants involved in the control group; since no differences were observed between the groups, there is a possibility that walking may be effective [12, 13]. Fourth, the baseline level of physical activity identified in both groups in our study was predominantly low to moderate; these patients may have required a higher level of intensity of exercise to produce a change in LOS [16, 17].

Other factors that could have affected the LOS in our study are the surgical time of fewer than 2 h, the age of patients below 59 years old, mostly women, and the majority living with their families. It has been described that early discharge to home is perceived for patients as early recovery and a restoration of their social network, an early return to work, and early ambulation may be more accelerated in some demographic groups like those that need to resume their functions promptly [17, 18].

The significant adherence to the intervention group's physical activity six months after hospital discharge is consistent with other results identified in non-surgery studies aimed to motivate behavior changes and improve health outcomes [18, 19]. We consider a potential explanation for this finding. Previous studies have shown that patients' significant adherence to walking was associated with the type of health provider that gave the prescription or the class of motivational intervention provided by health workers [20, 21]. In our study, the physician provided the prescription, an expert in rehabilitation, in a written form to establish a medical order that had to be carried out. It may happen that weekly reinforcements provided to the intervention group before surgery by their physician had motivated the patients to continue walking after discharge.

### Implementation for practice and research

The standardized indication of walking 150 min/week is based on physical activity recommendations determined by the exercise guidelines aimed at adults and the elderly, which indicates some benefits in the pre-operatory phase, like reducing BMI [22]. However, these guidelines still do not contain precise recommendations about the population that would benefit more from walking and the frequency/ intensity of the exercises that could improve clinical outcomes when applied before surgery [7, 22, 23]. A significant impact of walking before surgery can be observed if implemented during a time greater than 4 or 6 weeks, providing reinforcements of walking during the post-operatory period, as shown in some studies [12]. Hence, we do not know the best frequency and intensity of walking that would improve functional capacity before surgery, impacting LOS and complications after surgery.

Controlled clinical trials are the best epidemiological design to assess new interventions and medications' effectiveness and safety. Despite being studies with random assignment, clinical trials have limitations that can diminish the clinical validity of these and their applicability in certain groups of populations. Limitations in our study can be related to; first, recall bias of the patients in the intervention group to remember the time or the intervention despite the reinforcement calls provided weekly to them. Second, although we identified significant differences in compliance of walking before surgery by users of accelerometers in favor of the intervention group compared to the control group, the restricted number of accelerometers available limited a complete validation of the intervention's adherence. Third, the use of a diary to self-report the walking activity in consequence. The last limitation is related to our healthcare system's structure, which often delays information regarding the final authorization for the surgery dates. This factor may have affected the length of waiting times producing significant variability in pre-surgical times, affecting the intervention's efficacy.

## Conclusions

Our study is the first clinical trial evaluating the impact of walking before non-cardiac surgery in the length of stay, early ambulation, and complications after surgery. In conclusion, the prescription of a pre-surgical standardized 150-min walk per /week before non-cardiac surgery had no significant effect on length of stay or early ambulation and complications after surgery. The results do not support the use of simple walking prescription before non-cardiac surgery for reducing the length of hospital stay in adults.

The results become a crucial element for further investigation.

## Data Availability

The datasets used and analyzed during the current study are available from the corresponding author on Mendeley repository (10.17632/ft7r4wrnt3.1).

## Abbreviations

LOS: Length of hospital stay
PAMP: Promoting ambulation project
MET: Metabolic equivalent (MET), amount of oxygen consumed while sitting at rest and is equal to 3.5 ml O2 per kg body weight × min.
IPAQ: International Physical Activity Questionnaire
AMI: Acute myocardial infarction
CVA: Cerebrovascular accident
DVT: Deep venous thrombosis
BMI: Body Mass Index
CARDIECOL PROGRAM: Knowledge and action to reduce cardiovascular disease in Colombia
